# LGR5 is associated with tumor aggressiveness in papillary thyroid cancer

**DOI:** 10.18632/oncotarget.5330

**Published:** 2015-09-25

**Authors:** Gregory Michelotti, Xiaoyin Jiang, Julie Ann Sosa, Anna Mae Diehl, Brittany Bohinc Henderson

**Affiliations:** ^1^ Division of Gastroenterology, Duke University Medical Center, Durham, North Carolina, USA; ^2^ Department of Pathology, Duke University Medical Center, Durham, North Carolina, USA; ^3^ Department of Surgery, Duke University Medical Center, Durham, North Carolina, USA; ^4^ Division of Endocrinology, Diabetes and Metabolism, Wake Forest University, Winston-Salem, North Carolina, USA

**Keywords:** R-spondin, Wnt, β-catenin, metastasis, BRAFV600E

## Abstract

**PURPOSE:**

Leucine-rich repeat-containing G-protein-coupled receptor 5 (LGR5) is a cancer stem cell marker and a down-stream target in Wnt/β-catenin signaling. In human papillary thyroid cancer (PTC), over activation of Wnt/β-catenin has been associated with tumor aggressiveness.

**PATIENTS AND METHODS:**

Using established human cell lines (TPC-1, KTC-1, Nthy-ori-3–1), we report *LGR5 and* R-spondin *(RSPO1–3)* overexpression in PTC and manipulate *LGR5* and Wnt/β-catenin signaling via both pharmacologic and genetic interventions. We test the association of LGR5 tumor expression with markers of PTC aggressiveness using a Discovery Cohort (*n* = 26 patients) and a Validation Cohort (*n* = 157 patients). Lastly, we explore the association between LGR5 and the BRAFV600E mutation (*n* = 33 patients).

**RESULTS:**

Our results reveal that LGR5 and its ligand, RSPO, are overexpressed in human PTC, whereby Wnt/β-catenin signaling regulates LGR5 expression and promotes cellular migration. In two separate cohorts of patients, LGR5 and RSPO2 were associated with markers of tumor aggressiveness including: lymph node metastases, vascular invasion, increased tumor size, aggressive histology, advanced AJCC TNM stage, microscopic extra thyroidal extension, capsular invasion, and macroscopic invasion. As a biomarker, LGR5 positivity predicts lymph node metastasis with 95.5% sensitivity (95% CI 88.8%-98.7%) and 61% specificity (95% CI: 48.4%–72.4%) and has a negative predictive value (NPV) of 91.3% (95% CI 79.2%–97.5%) for lymph node metastatic disease. In human PTC, LGR5 is also strongly associated with the BRAFV600E mutation (*p* = 0.005).

**CONCLUSION:**

We conclude that overexpression of LGR5 is associated with markers of tumor aggressiveness in human PTC. LGR5 may serve as a future potential biomarker for patient risk stratification and loco regional metastases in PTC.

## INTRODUCTION

The Wnt/β-catenin signaling pathway plays an important role in stem cell proliferation, tissue differentiation, and cellular polarity [1–2]. Dysregulation of Wnt/β-catenin signaling contributes to the development of human papillary thyroid cancer (PTC), potentiating cancer stem cell proliferation, disruption of thyroid microarchitecture, tumor dedifferentiation, extra capsular spread, and angioinvasion [3–5]. Recently, theories on thyroid cancer biogenesis have migrated from the classical explanation (i.e “multi-hit” genetic mutations and environmental exposures) to the promotion of tumor-genesis by proliferation of cancer stem cells (CSCs) [6–7]. In the CSC hypothesis, embryonic cells in the thyroid gland (i.e. thyroblasts and prothyrocytes) are susceptible to tumor formation and accumulate mutations that give rise to thyroid cancer.

Leucine-rich repeat-containing G-protein-coupled receptor 5 (LGR5) is specifically expressed on CSCs and is a known downstream target gene of the Wnt/β-catenin pathway [8–12]. The LGR5 receptor is structurally similar to the thyroid-stimulating hormone (TSH) receptor [13], consistent with a role for LGR5 in thyroid pathophysiology. Recently, LGR5 has been shown to bind with high affinity to a family of secreted growth factor ligands known as R-spondins (RSPO1–4) [14]. The four paralogs share 40–60% pairwise amino acid sequence identity and are predicted to share substantial structural homologies. A characteristic feature of all four RSPO members is their ability to activate LGR5-frizzled lipoprotein receptor-related protein 5 and 6 (LRP5/6) binding and enhance Wnt-mediated β-catenin activation. [15–20].

In recent years, multiple studies have demonstrated that LGR5 is overexpressed in various types of tumors, including colorectal [21], ovarian [22], hepatocellular [23], basal cell [24], and esophageal cancers [25]. In several of these cancers, high LGR5 expression is associated with the initiation, invasion, and metastasis of tumors, suggesting a potential role for LGR5 in tumor genesis [26, 27]. Still, controversial literature exists claiming that LGR5 is a negative regulator of tumorigenicity [28, 29]. We tested the hypothesis that overexpression of the CSC marker, LGR5, is associated with markers of thyroid tumor aggressiveness, including cellular migration, features of histological aggressiveness, loco-regional metastases, and presence of the BRAFV600E mutation.

## RESULTS

### LGR5 and RSPO are overexpressed in human papillary thyroid cancer

**Figure 1:**
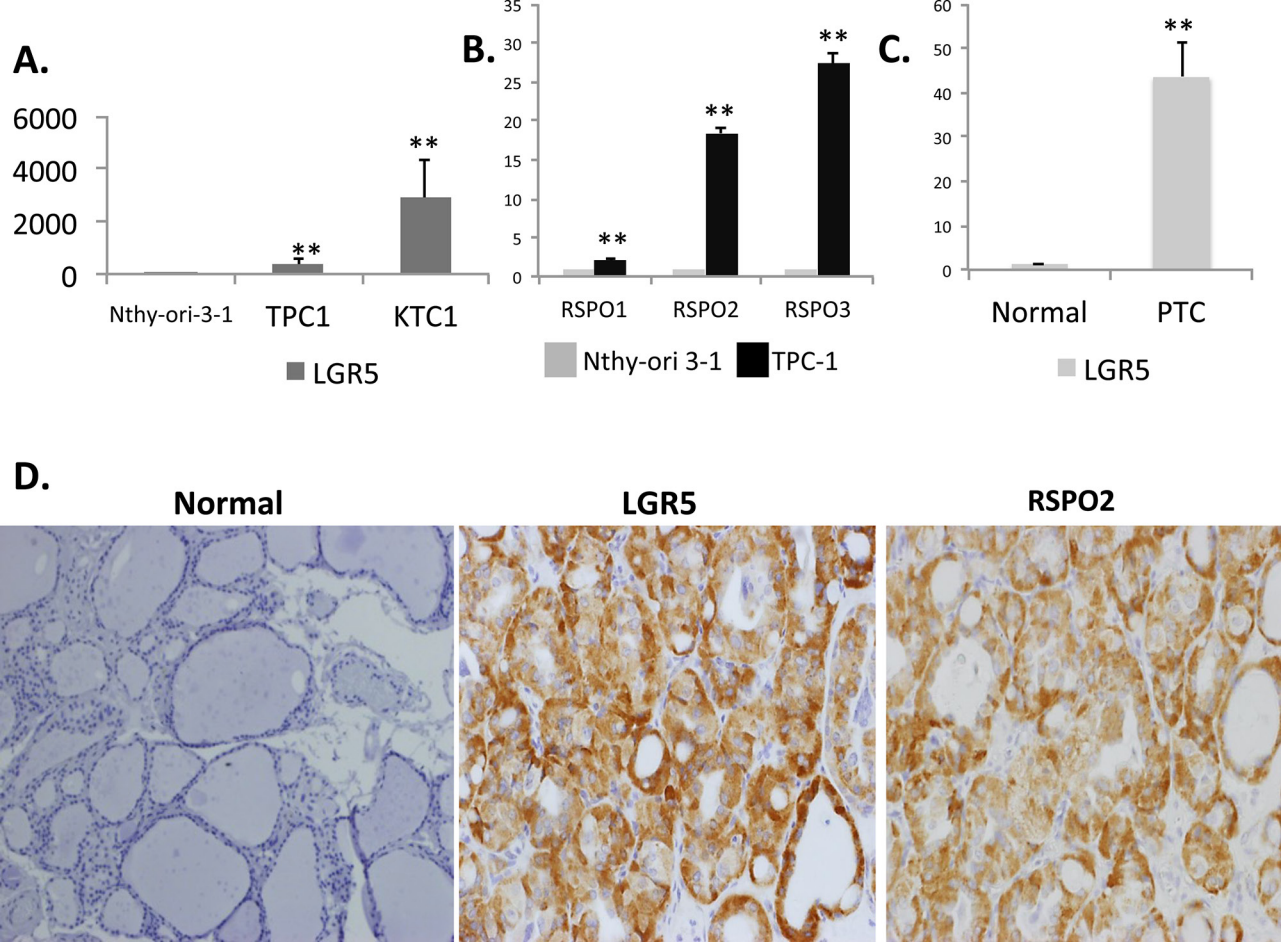
LGR5 and RSPO are induced in human thyroid cancer Total RNA was isolated from Nthy-ori 3–1, a normal human thyroid cell line, and PTC cell lines TPC-1 and KTC-1. Expression of **A.** LGR5 and **B.** RSPO1–3 were quantified by qRT-PCR. Results are normalized to β-actin gene expression and expressed as mean fold-over Nthy-ori-3-1 for each gene (*n* = 3 independent isolations). **C.** *LGR5* mRNA expression in normal frozen human tissue samples (*n* = 4 samples) and papillary thyroid cancer samples (*n* = 6 samples). **D.** Representative immunohistochemistry confirming elevated protein expression of both LGR5 and RSPO2 in patients with pathologically characterized PTC relative to normal thyroid tissue (magnification x20). Significance *p* < 0.05**.

**Figure 2:**
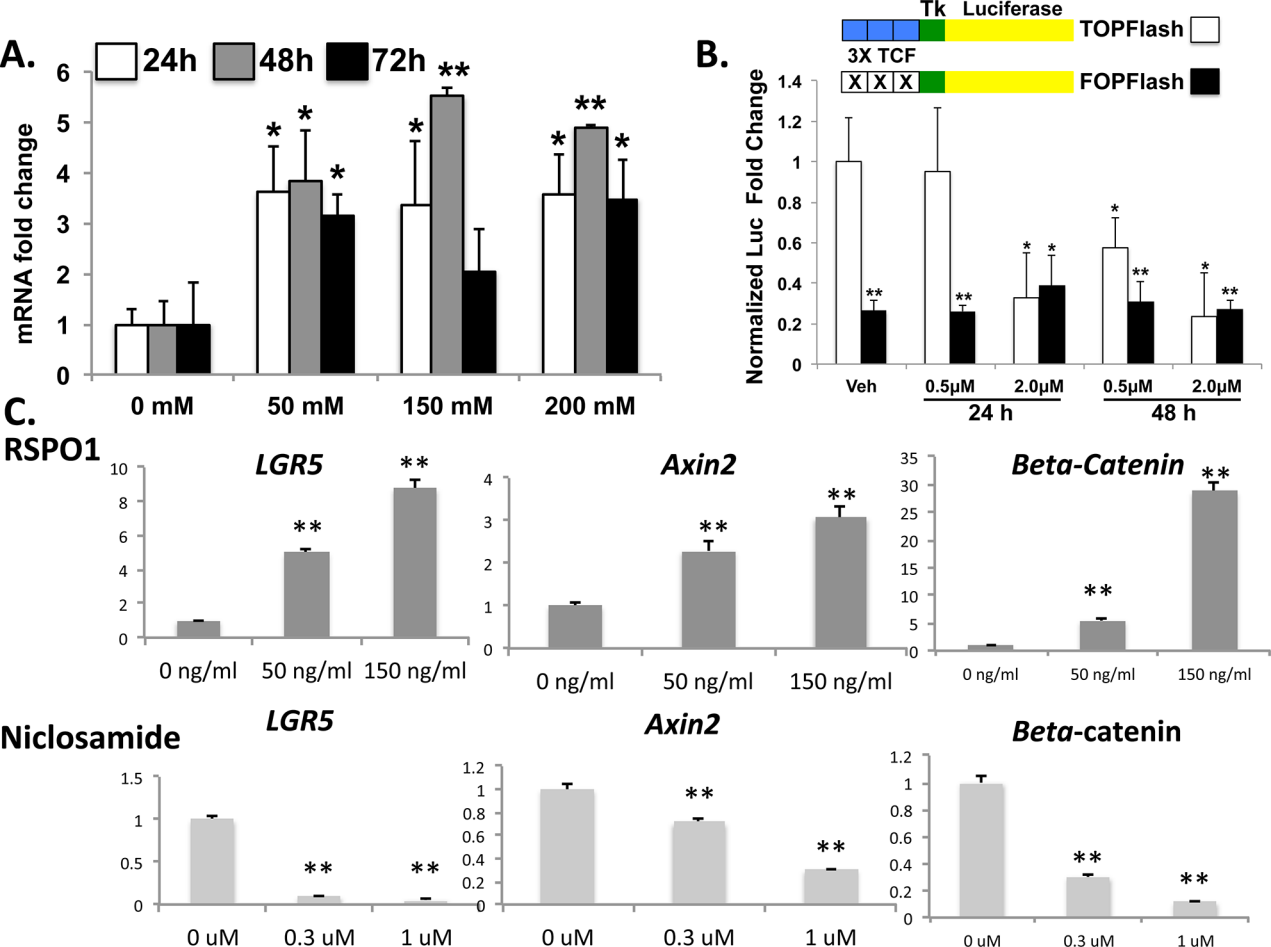
TPC-1 cells demonstrate native Wnt/β-catenin signaling with pharmacologic manipulation **A.** TPC-1 cells were treated either with increasing concentrations of recombinant RSPO1 for 24, 48 and 72 hours and LGR5 expression was examined by qRT-PCR. Results represent the mean of three independent experiments expressed as fold over vehicle (VEH) control for each time point. *P < 0.05; **P ≤ 0.01 **B.** TPC-1 cells were transfected with TOPFLASH or FOPFLASH as indicated and treated with either vehicle (DMSO), 0.5 uM, or 2.0 uM niclosamide for either 24 or 48 hours. Renilla luciferase plasmid was as normalization control and results are expressed as mean fold-change from baseline (vehicle; *n* = 2). **C.** TPC-1 cells were treated with compounds to either inhibit (niclosamide) or activate (RSPO) the Wnt pathway. TPC-1 cells were treated either with recombinant RSPO1 or niclosamide at the indicated doses for 48 hours and mRNA expression levels for *LGR5, Axin2* and *β-catenin* were quantified. Values were normalized to β-actin housekeeping gene expression and expressed as the fold over DMSO control (mean ± SEM; *n* = 3). Significance *p* < 0.05**.

Using established *in vitro* models and human samples, we found that the LGR5 receptor and its R-spondin ligands (RSPO 1–3) are overexpressed in human PTC [Figures 1A–1D]. Consistent with published literature stating that RSPOs are functional ligands for the LGR5 receptor, we confirm dose-dependent up-regulation of LGR5 in the presence of RSPO ligand [Figures 2A, 2C]. In addition to cellular models of PTC, frozen human thyroid tumors expressed significantly more LGR5 by mRNA and protein analysis than adjacent benign frozen thyroid tissue samples (*n* = 4 normal tissue samples, *n* = 6 tumor samples) [Figure 1C]. Immunohistochemical staining of human PTC demonstrated variable expression of LGR5 and RSPO2 between patient samples, with some patients strongly expressing the protein and others completely devoid of expression [Supplementary Figures S1 and 2]. In several cases, there was striking intra-tumoral heterogeneity [Supplementary Figure S3].

### LGR5 expression potentiates Wnt/β-catenin signaling in papillary thyroid cancer

RSPO-LGR5 signaling has been shown to potentiate Wnt/B-catenin signaling in other tissues, particularly in colorectal carcinoma (CRC). We effectively demonstrated that treatment of *in vitro* human PTC cell lines with RSPO ligand enhanced *LGR5, B-catenin and Axin2* mRNA expression, whereas treatment of the cell lines with Wnt antagonist, niclosamide, effectively down-regulated Wnt target genes *and LGR5* expression [Figures 2C]. Direct evidence for manipulation of human PTC cell lines in Wnt/β-catenin signaling was confirmed by TOP/FOP flash assay after treatment of these cell lines with niclosamide, and cell death was observed in a dose-dependent manner by both visual observation of floating cells and by viability count using Trypan Blue and a hemocytometer (*observational data not shown*) [Figure 2B]. Results were similar in all TPC-1, KTC-1 and Nthy-ori-3-1 *in vitro* models.

### LGR5 is important in cellular migration and proliferation

**Figure 3:**
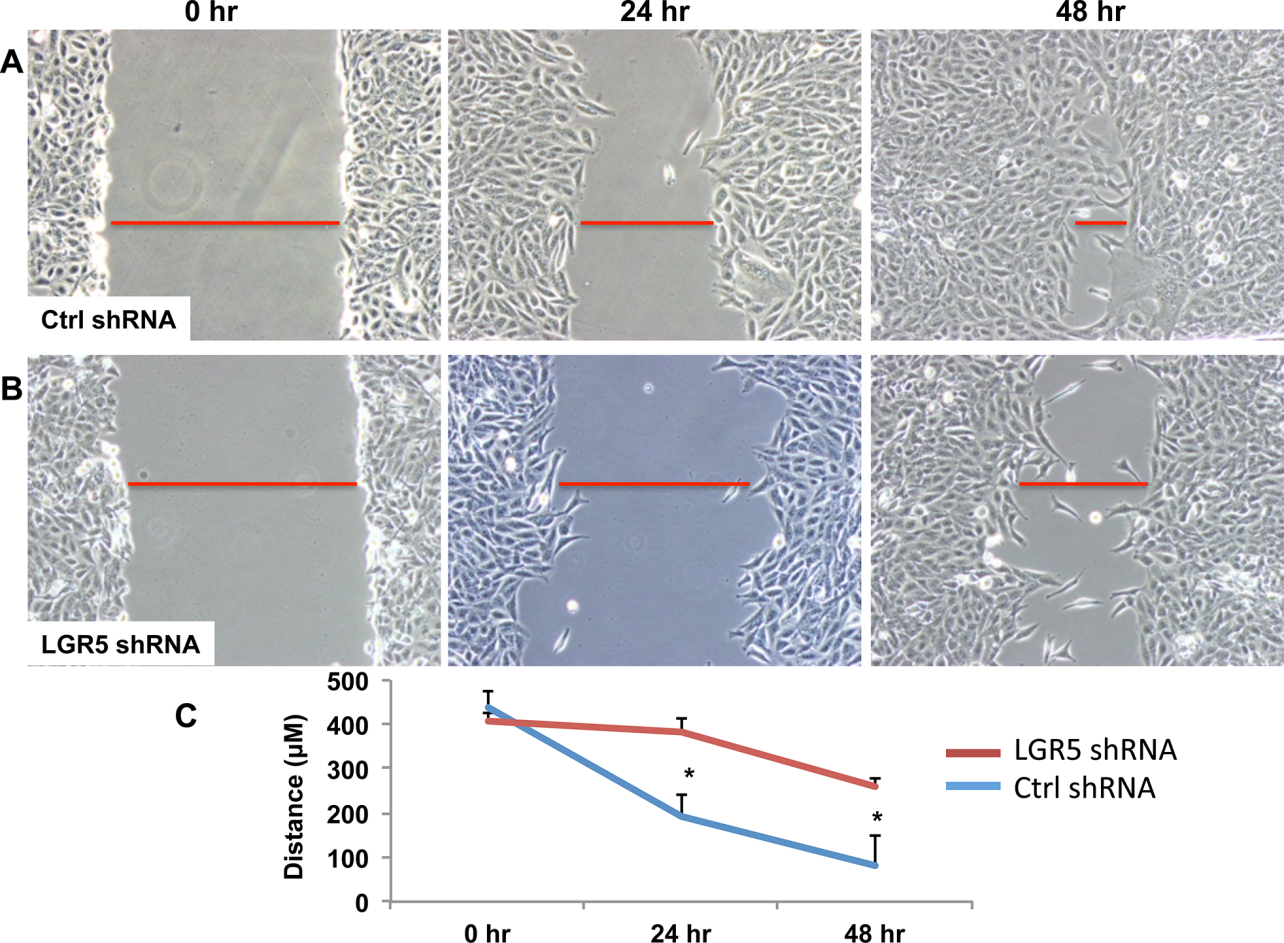
shRNA-mediated decrease in LGR5 expression results in inhibition of cell migration TPC-1 clonal cells harboring stable expression for either non-targeting (Ctrl) shRNA **A.** or targeting human LGR5 shRNA **B.** were subjected to a wound healing assay for 24 and 48 h. Representative images are shown. Leading edges of repairing cell monolayers are indicated by red bars. Briefly, cells were grown to 80% confluence at which time cells were removed with a P200 tip. Images were then acquired at 0 h, 24 h, and 48 h and analyzed by Olympus software to measure ability of the cells to repair the wound. **C.** Cellular migration was quantified and represented graphically with results expressed as mean ± SEM of three independent measurements. Significance *p* < 0.05*.

Overexpression of LGR5 constructs in human cell lines had no effect on cellular phenotype or proliferation, likely due to overexpression at baseline. Conversely, silencing of *LGR5* by shRNA showed impressive effects on reduced cellular migration by scratch assay, suggesting that it may be important in tumor migration and aggressiveness [Figures 3A–3C, Supplementary Figures S4 and S5]. CCK8 assay demonstrated no effects of shRNA on cellular proliferation in these cell lines [*data not shown*].

### LGR5 overexpression in human PTC is associated with tumor aggressiveness and metastatic disease

In three separate PTC human cohorts treated at Duke from 2005–2013, we demonstrated that LGR5 positivity was highly associated with markers of tumor aggressiveness. Our Discovery Cohort (*n* = 26 patients) demonstrated that LGR5 positivity was significantly associated with male gender (*p* = 0.009), vascular invasion (*p* = 0.015), and lymph node positivity (*p* < 0.0001) [Table 1]. In LGR5-positive tumors associated with metastatic lymph nodes, tumor foci in lymph nodes were similarly LGR5 positive [Supplementary Figure S6]. In a small cohort of tumors that showed heterogeneous LGR5 expression (*n* = 6 tumors), corresponding lymph node metastases were all LGR5 positive, suggesting that the LGR5 population of tumoral cells were migratory to loco regional sites. Because PTC is classically associated with lymph node metastasis in up to 60% of patients at presentation, we wished to ensure that we were evaluating patients who had a representative sample of nodes dissected at initial surgery. Therefore, in our Validation Cohort (*n* = 157 patients), we ensured that all patients underwent total thyroidectomy with at least 5 lymph nodes sampled at surgery. In this population, we found significant association of LGR5 with markers of tumor aggressiveness, including primary tumor size (*p* = 0.010), lymph node metastases, and N stage (*p* < 0.0001), number of nodal metastases (< 0.0001), T Stage (*p* = 0.0104), vascular invasion (*p* < 0.0001), microscopic extra-thyroidal extension (*p* < 0.0001), capsular invasion (*p* = 0.0140), and macroscopic invasion (*p* = 0.007) [Table 2]. LGR5 positivity was associated with male gender, which is a known risk factor for more aggressive tumors. LGR5 also was associated with more aggressive histology, particularly the tall cell variant of PTC (*p* = 0.0008). Multifocality was associated with LGR5 negative dominant tumors. As a biomarker, LGR5 positivity in the Validation Cohort predicted the presence of lymph node metastasis with a 95.5% sensitivity (95% CI 88.8%–98.7%) and 61% specificity (95% CI: 48.4%–72.4%); it had a negative predictive value (NPV) of 91.3% (95% CI 79.2%–97.5%) for metastatic disease to lymph nodes. In the validation cohort, RSPO2 positivity strongly correlated with LGR5 tumor expression (*p* < 0.0001), with 80% of LGR5-positive tumors also staining positive for ligand RSPO2 [Supplementary Figure S2]. RSPO2-positivity was strongly associated with nodal metastases (*p* = 0.029). Double-positive tumors (for LGR5 and RSPO2) were significantly associated with nodal metastases (*p* < 0.0001), number of lymph nodes positive (*p* = 0.0093), lymphovascular invasion (*p* = 0.0491), and extrathyroidal extension (*p* = 0.0355).

**Table 1:** The Discovery Cohort (*n* = 26 patients)

Discovery Cohort Characteristics	LGR5 – (*n* = 10 patients)	LGR5 + (*n* = 16 patients)	*p* value
Male Sex	12.5%	66.67%	**0.009**
Age (years)	47.6	49.0	**0.794**
Tumor Size (mean size +/− std dev)	2.67 cm (+/−2.1 cm)	2.08 cm (+/−1.08 cm)	**0.860**
PTC Variant	Classical = 6 (60%) Follicular Variant = 3 (30%) Classical + Follicular Variant = 1 (10%)	Classical = 9 (56.25%) Follicular Variant = 3 (18.75%) Classical + Follicular Variant = 4 (25%)	**0.567**
Lymph Node Positivity	0%	81.3%	**<0.0001**
Multifocality	20%	50%	**0.126**
Encapsulated	0%	21.43%	**0.118**
Vascular Invasion	0%	33.33%	**0.015**
Extrathyroidal Extension	0%	13.33%	**0.142**
Positive Margins	10%	25%	**0.345**

Patient and tumor characteristics are reported along with LGR5 tumor status by immunohistochemistry. Significance is highlighted in red and is ***p*** < 0.05**

**Table 2:** The Validation Cohort (*n* = 157 patients)

Validation Cohort Characteristics	LGR5 – (*n* = 48 patients)	LGR5 + (*n* = 109 patients)	*p* value
Male Sex	19.6%	26.1%	<0.0001**
Age (years)	44.5 (+/−14.1)	44.0 (+/−16.6)	**0.851**
Tumor Size (cm) (mean size +/− std dev)	1.6 (+/−1.3)	2.3 (+/−1.6cm)	0.010*
PTC Variant	Classical= 30.8% Follicular Variant = 69.2% Tall cell variant = 0%	Classical = 68.6% Follicular Variant = 25.7% Tall cell variant = 8.6%	0.0008**
Lymph Node Positivity	8.3%	75.2%	<0.0001**
Lymph Node Number	0.7 +/−2.6	6.2 +/−8/1	<0.0001**
Multifocality	34.8%	31.5%	0.0154*
T Stage	T1–2: 93.5% T3–4: 6.5%	T1–2: 83.7% T3–4: 16.2%	0.0104*
Vascular Invasion	8.7%	33.6%	<0.0001**
Extrathyroidal Extension	2.2%	26.4%	<0.0001**
Capsular Invasion	10.9%	14.5%	0.0140*
Macroscopic Invasion	0%	2.8%	0.007**

Patient and tumor characteristics are reported along with LGR5 tumor status by immunohistochemistry. Significance is highlighted in red and is ***p*** < 0.05**

### LGR5 is more highly expressed in PTC that harbors the BRAFV600E mutation

**Figure 4:**
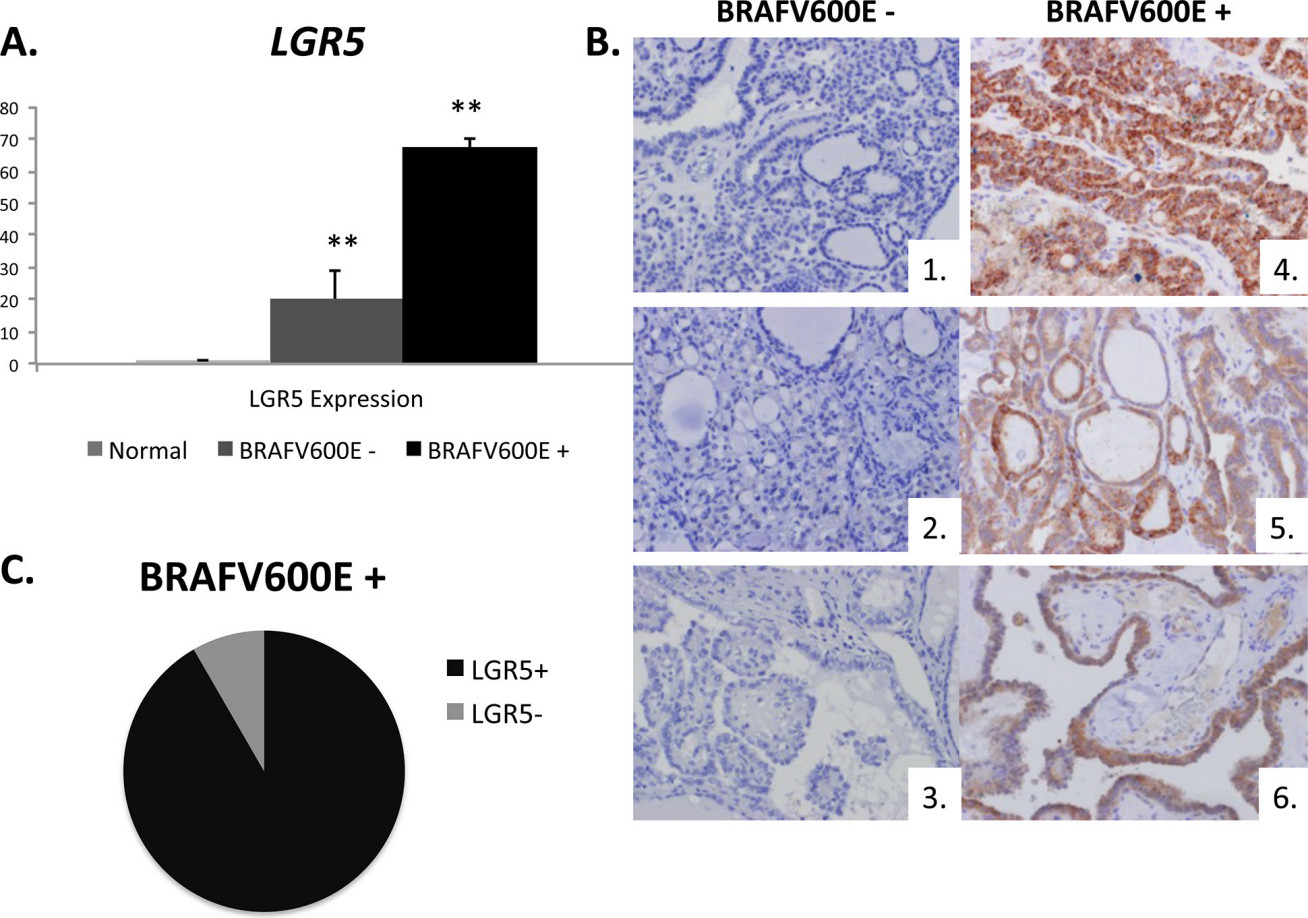
LGR5 tumoral positivity is associated with the BRAFV600E mutation **A.** Expression of *LGR5* in frozen normal human thyroid tissue (*n* = 4 normal samples) and in BRAFV600E tumor negative and positive samples (*n* = 3 samples/group). **B.** Representative IHC tumor samples for LGR5 (brown DAB staining) in BRAFV600E negative patients (1–3) and BRAFV600E positive patients (4–6). **C.** In a cohort of 33 human patients with known BRAFV600E mutational status, LGR5 positivity was strongly associated with the BRAFV600E mutation (*p* = 0.005**). In those who were BRAFV600E positive, 92% were also LGR5 positive (black) while 8% were LGR5 negative (grey). Significance *p* < 0.05**.

Because the BRAFV600E mutation is known to be associated with higher risk of recurrence, more aggressive histology, and increased risk for lymph node and distant metastases [32], we assessed whether there was association between LGR5 positivity and the BRAFV600E mutation. Using frozen human tissue samples, we measured *LGR5* mRNA expression in normal thyroid tissue and in PTC tissue with and without the BRAFV600E mutation (*n* = 4 patient samples/group). LGR5 *mRNA* expression was 3-fold higher in BRAFV600E mutation positive tumors than in tumors with wild type expression [Figure 4A]. In a separate cohort of 33 human patients with known BRAFV600E mutational status, LGR5 positivity was strongly associated with BRAFV600E mutational positivity by protein analysis (*p* = 0.005), strengthening the conclusion that LGR5 tumor positivity is associated with markers of tumor aggressiveness [Figure 4B]. In tumors that harbored the BRAFV600E mutation, 92% also were found to be LGR5 positive [Figure 4C].

## DISCUSSION

Our data is the first to report that LGR5 and its ligand, RSPO2, are associated with tumor aggressiveness, lymph node metastases, and Wnt/β-catenin activation in human papillary thyroid cancer.

Both the TPC-1 and KTC-1 cell lines harbor mutations that up-regulate MAPK/ERK signaling cascades (RET/PTC1 and BRAFV600E, respectively). Previous studies have suggested that up-regulation of MAPK/ERK is associated with β-catenin stabilization and TCF-mediated nuclear transcription. The exact mechanism for this synergistic process is yet unclear, but our data supports an existing body of literature suggesting that simultaneous up-regulation of MAPK/ERK and Wnt/β-catenin promotes tumor migration and metastases [33, 34].

Our findings also support a growing body of literature associating LGR5 with aggressiveness in other cancer subtypes including: colorectal, gastric, and cervical cancers, as well as malignant glioma [35–42]. In these tumors, LGR5 has been associated with increased cellular proliferation, resistance to chemotherapy, and compromised patient survival [38, 39]. Supporting evidence that LGR5 is an important marker of tumor aggressiveness has emerged particularly in the arena of colorectal cancer. Similar to our findings, evaluation of human colorectal tumors by IHC demonstrated that LGR5 over-expression was more frequently found in the metastatic lymph nodes and distant metastases when compared with primary colorectal cancer tissue. Additionally, cancer cells in the invasive front presented stronger LGR5 immunoreactivity than that at tumor center (*P* < 0.05) [39]. Three larger human studies (*n* = 366 patients, *n* = 192 patients, *n* = 2139 patients) similarly found that LGR5 colorectal cancer tumor expression was significantly associated with higher American Joint Committee on Cancer (AJCC) staging, increased Ki67 indices, advanced histological grade, increased depth of invasion, presence of nodal and distant metastases, and was independently associated with compromised overall patient survival [41, 42]. Taken together, these findings suggest that LGR5 may be both a prognostic marker and a potential target for patients with aggressive and metastatic tumors.

PTC is associated with low overall mortality but high increased morbidity and risk for loco regional recurrence [43]. Therefore, although patients are usually not at high risk for death, they are subject to repeat ultrasound surveillance, serologic and radiographic testing, and accumulating health care costs as part of lifelong monitoring for demonstration of persistent or recurrent disease. An overwhelming majority of recurrences are found in loco regional lymph nodes, potentially prompting higher-risk remedial surgery, increased iodine-131 (I-131) cumulative doses, and as a result increased risk of patient morbidity. On average, 7% of all PTC patients have persistent PTC in the first year following treatment, and another 2% of patients recur over 8–10 years of follow-up; nevertheless, many patients undergo a lifetime of extensive surveillance testing. Risk for recurrence is strongly associated with tumor risk stratification largely based on tumor histology, with only 2.5% of low-risk patients but up to 70% of high-risk patients found to have persistent disease within the first postoperative year [42]. Because current treatment methods cannot predict risk stratification pre-operatively (prior to tissue analysis), extent of initial surgery is currently left to physician discretion (based on surgeon preference, tumor size, patient age, and highly variable operator-dependent preoperative ultrasonography) [39]. Pre-operative dilemmas of not knowing the exact tumor size prior to surgical resection, inability to assess for markers of tumor aggressiveness, and compromised ultrasonographic assessment of lymph node status in the central neck compartment prior to thyroidectomy (because of the overlying thyroid gland) can severely restrict the ability to make an informed decision on the appropriate extent of surgery [44, 45]. These challenges are reflected in discordance among surgical guidelines internationally; the American Thyroid Association, for example, endorses total or hemi thyroidectomy without prophylactic node dissection [44], while the Japanese guidelines suggest prophylactic central neck lymph node dissection +/− lateral neck lymph node dissection at the time of primary surgery due to the high incidence of microscopic lymph node metastases with PTC (upwards of 60–70% at diagnosis) [45]. Therefore, it would be beneficial to be able to accurately predict preoperatively tumoral behavior and risk for metastatic disease in order to: (1) reduce postoperative long-term surveillance in low risk patients and (2) perform the best initial surgery, thereby minimizing persistence/recurrence and repeat medical and surgical intervention in intermediate and high risk patients.

We demonstrated in two separate cohorts of PTC patients that LGR5 positivity is associated with aggressive histological characteristics such as lymphovascular invasion, increased tumor size, microscopic extra thyroidal extension, capsular invasion, macroscopic invasion, and more aggressive tumor type. It is associated with increased TNM stage, increased number of metastatic lymph nodes, and BRAFV600E positivity. LGR5 positivity is also associated with male gender, which is a demographic characteristic associated with, on average, more aggressive tumor behavior. Interestingly, established markers of PTC recurrence including ETE and more than 5 metastatic lymph are also associated with LGR5 and RSPO2 positivity. Further research is needed to determine whether LGR5 overexpression may be beneficial as a preoperative biomarker and tool for risk stratification. Tumor positivity may help direct surgical approach and postoperative surveillance. Additionally, LGR5 may serve as a novel target for the management of aggressive PTC. Accordingly, there is a need to better understand the role that LGR proteins and RSPO proteins play in carcinogenesis and metastasis. Future studies will focus on delineating the mechanism for enhanced LGR5-induced tumoral migration and development of LGR5 as a pre-operative biomarker and as a prognostic marker of disease-specific morbidity/recurrence in human PTC.

## MATERIALS AND METHODS

### Human cell lines and cell culture

Human papillary thyroid cancer cell lines (TPC-1 and KTC-1) were grown in RPMI1640 media (Sigma-Aldrich). The immortalized normal human thyroid cell line (Nthy-ori-3-1, ATCC) was cultured in RPMI 1640 supplemented with 2 mM glutamine and 10% fetal bovine serum (FBS) (*Sigma-Aldrich*). All media were supplemented with 10% heat-inactivated FBS (*Invitrogen, Carlsbad, CA, USA*) and 1% penicillin-streptomycin (10,000 units penicillin and 10 mg streptomycin per mL in 0.9% NaCl, *Sigma-Aldrich*). Culturing was carried out at 37°C with 5% carbon dioxide.

### Drug manipulations

Wnt signaling was assessed at baseline in Nthy-ori-3-1, TPC-1 and KTC-1 cell lines. Human cell lines were treated with recombinant human R-Spondin1 (RSPO1, *R&D Systems, 4645-RS-025*) at varying concentrations in a dose-dependent manner (0 ng/ml, 50 ng/ml, and 150 ng/ml) and harvested after 48 hours for mRNA and protein. Additionally, cell lines were treated with increasing doses of niclosamide (0 μM, 0.3 μM, and 1 μM), a Wnt/β-catenin antagonist, and harvested at 48 hours. All drug doses were consistent with published literature utilizing oncogenic cell lines [30].

### Quantitative real-time PCR (qRT-PCR)

RNA was isolated from individual cell lines and frozen human thyroid tissue using Direct-zol RNA Miniprep kit (*ZYMO, R2052*) and quantified by Nanodrop 2000 (*Thermo Scientific*). RNA was reverse transcribed to cDNA using random primer and Superscript RNase Reverse Transcriptase (Invitrogen, Carlsbad, CA, USA) and amplified using the StepOne Plus real-time PCR platform (*ABI/Life Technologies*). qRT-PCR was performed with denaturation at 95°C for 3 minutes, followed by 40 cycles of denaturing at 95°C for 10 seconds and annealing-extension at the optimal primer temperatures for 60 seconds. Target gene threshold cycles (Ct) were analyzed according to the 2-ΔΔCt method relative to human beta actin housekeeping gene. All analyses were performed in triplicate. Primer sequences can be found in Supplementary Material.

### TOP/FOP-flash assay

We used a TOP/FOP-Flash reporter assay reflecting beta-catenin activation of TCF-LEF mediated transcription and Wnt pathway activity, as described [31]. Briefly, TOP-Flash and FOP-Flash plasmids (2 μg) were transfected into tumor cells with JetPrime (Polyplus Transfection) in 24-well plates and treated with either vehicle (DMSO) or niclosamide (1 μM, Sigma). pTK-RL plasmid encoding Renilla luciferase (0.1 μg, Promega) was used as reference plasmid. The activity of both luciferase reporters was determined at 48 hours after transfection using the Dual Luciferase Assay kit (Promega, Madison, WI, USA), according to the manufacturer's instructions. The TOP-Flash reporter activity is presented as the relative ratio of firefly luciferase activity to Renilla luciferase activity. All experiments were performed in triplicate.

### Overexpression and knockdown of LGR5

TPC-1 cells stably overexpressing either non-targeting (Control, Ctrl) or three distinct LGR5 targeting shRNAs (Origene) were isolated. One day prior to transfection of plasmids harboring unique LGR5 shRNA silencing sequences, TPC-1 cells were seeded at 50% confluency on 6-well tissue culture plate. 5 ng/ul of three distinct plasmids harboring LGR5 shRNAs (Origene; TF311747) were added with JetPrime transfection reagent to each well according to the manufacturer's protocol. Transfection efficiency was monitored by fluorescent microscopy to visualize red fluorescent protein expression of an integrated turboRFP element in the plasmid. Cells stably overexpressing LGR5-targeting shRNA were selected using puromycin antibiotic (Sigma) at a previously optimized concentration of 5 μg/ml. Clonal populations were isolated, expanded and maintained under puromycin selection.

### Cellular viability assays

Cell viability was measured using the Cell Counting Kit-8 (CCK8; Dojindo Molecular Technologies, Gaithersburg, Maryland, USA). Briefly, wild-type TPC-1 cells, and TPC cells stably expressing either non-targeting shRNA or LGR5 shRNA were plated and cell growth measured over 48 hours. Absorbance measurements were made by the FluoroStar Optima plate reader (BMG Labtech) at excitation filter 465 nm. Measurements were performed in triplicate.

### Wound-healing assay

A wound-healing assay was performed by scraping cell monolayers. Cells were plated at 80% confluency, and a wound was made using a P200 tip. A reference mark was created on the dish, and a time 0 image was acquired. Subsequent images were taken in the matched region at the indicated times, and the distance of the wound-healing area lacking cells was quantified (Olympus DP2-BSW software).

### Human studies

#### Clinical cohorts

Using the IRB-approved Duke Clinical and Pathologic Database conducted according to the principles expressed in the Declaration of Helsinki, we identified a discovery cohort of patients with PTC (*n* = 26 patients) and associated clinical-histologic characteristics. To confirm preliminary findings, we identified a validation cohort of patients (*n* = 157 patients) treated at Duke from 2005–2013 who had undergone total thyroidectomy with at least 5 lymph nodes sampled at surgery (*n* = 71 patients with N0 disease and 86 patients with N1 disease). Surgeries were performed by a variety of surgeons (*n* = 7) at our institution. A separate cohort of patients (*n* = 33 patients) with known BRAFV600E tumor mutational status was evaluated for association of this mutation with LGR5 positivity Figure 4.

### Immunohistochemistry

The immunohistochemical staining procedure was performed on unstained paraffin-embedded slides. Briefly, formalin-fixed and paraffin-embedded tissue sections (5 μm thick) were deparaffinized in xylene and rehydrated through descending concentrations of ethanol. Endogenous peroxidase activity was quenched using hydrogen peroxide (H_2_0_2_) for 14 minutes on all tissue specimens. In each case, a control slide was incubated with DAB substrate solution after rehydration to water to confirm quenching of endogeneous peroxidase activity after treatment with H_2_O_2_. After antigen retrieval was performed by heating in 10 mM citrate buffer (pH 6.0) + 0.05% TWEEN20 for 20 min, the sections were treated with protein block (*DAKO Envision, Carpinteri, CA*). Primary antibodies for LGR5 (*Origene, TA301323*, 1:500 dilution) and RSPO2 (*Origene, NBP1–81093* 1:500 dilution) were added to tissue sections and incubated overnight at 4°C. A secondary control was used for each run with omission of the primary. A horseradish peroxidase-conjugated rabbit or mouse secondary antibody was added for 60 min at room temperature, followed by 3,3′-diaminobenzidine (DAB) development (*DAB Substrate Chromogen System, Dako*) and hematoxylin and eosin (H&E) as per standard staining protocol. Slides were fixed and images obtained with the Olympus IX71 inverted microscope using the DP2-BSW Olympus image acquisition software system. LGR5 and RSPO2 tumor staining was classified into two categories: negative or positive expression. All slides were reviewed in triplicate in a blinded manner. Staining was performed on primary tumor samples (*n* = 208 total patients) and in a representative cohort of corresponding lymph node metastatic samples (*n* = 12 patients).

### Statistical analysis

A statistical analysis was performed using JMP software, version 10 (Cary, NC). For comparison among the groups, a Student's *t* test, Chi-Square test or a Fisher's Exact test followed by a *post hoc* Tukey test was performed, and *p* < 0.05 was defined as statistically significant. The data and error bars report the means ± SEM.

### Study approval

The Duke Institutional Animal Care and Use Committee and the Institutional Review Board approved all animal and human studies in compliance with Duke University Ethical and Research standards. Duke University conducts all research according to the Declaration of Helsinki principles.

## SUPPLEMENTARY FIGURES AND TABLE


