# Supplementary material for: LGR5 is associated with tumor aggressiveness in papillary thyroid cancer
Source: Oncotarget. 2015 Sep 25;6(33):34549–60. doi: 10.18632/oncotarget.5330 (PMC4741472; doi:10.18632/oncotarget.5330)
Supplement: Supplementary file 1 [file oncotarget-06-34549-s001.pdf]

SUPPLEMENTARY FIGURES AND TABLE

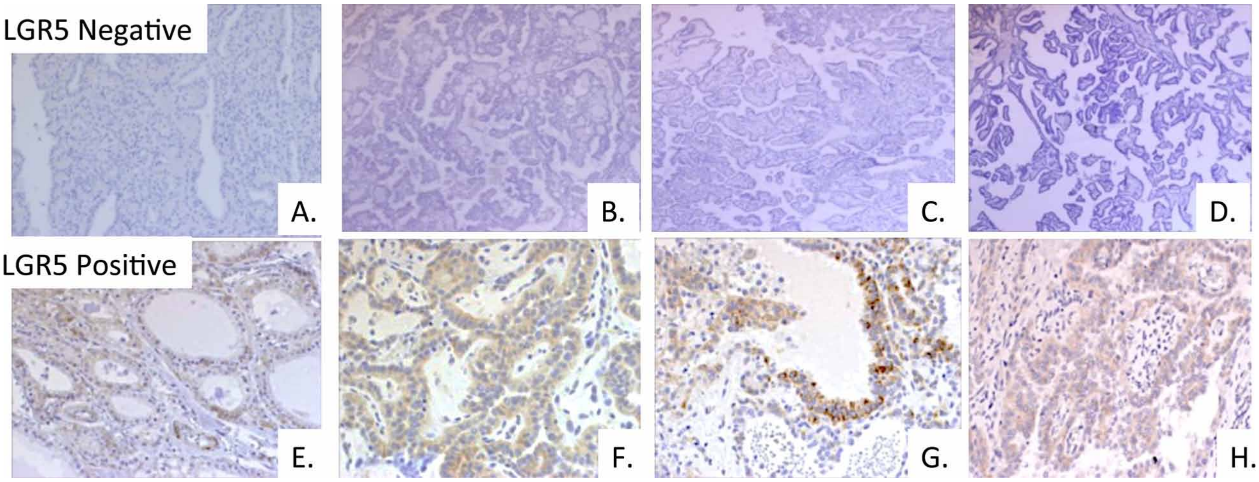

Supplementary Figure S1: Human thyroid tumors can be LGR5 positive or LGR5 negative. Representative IHC staining in several patients with LGR5 negative tumors **A–D.** and LGR5 positive tumors **E–H.** LGR5 protein expression is demonstrated in brown (magnification x20).

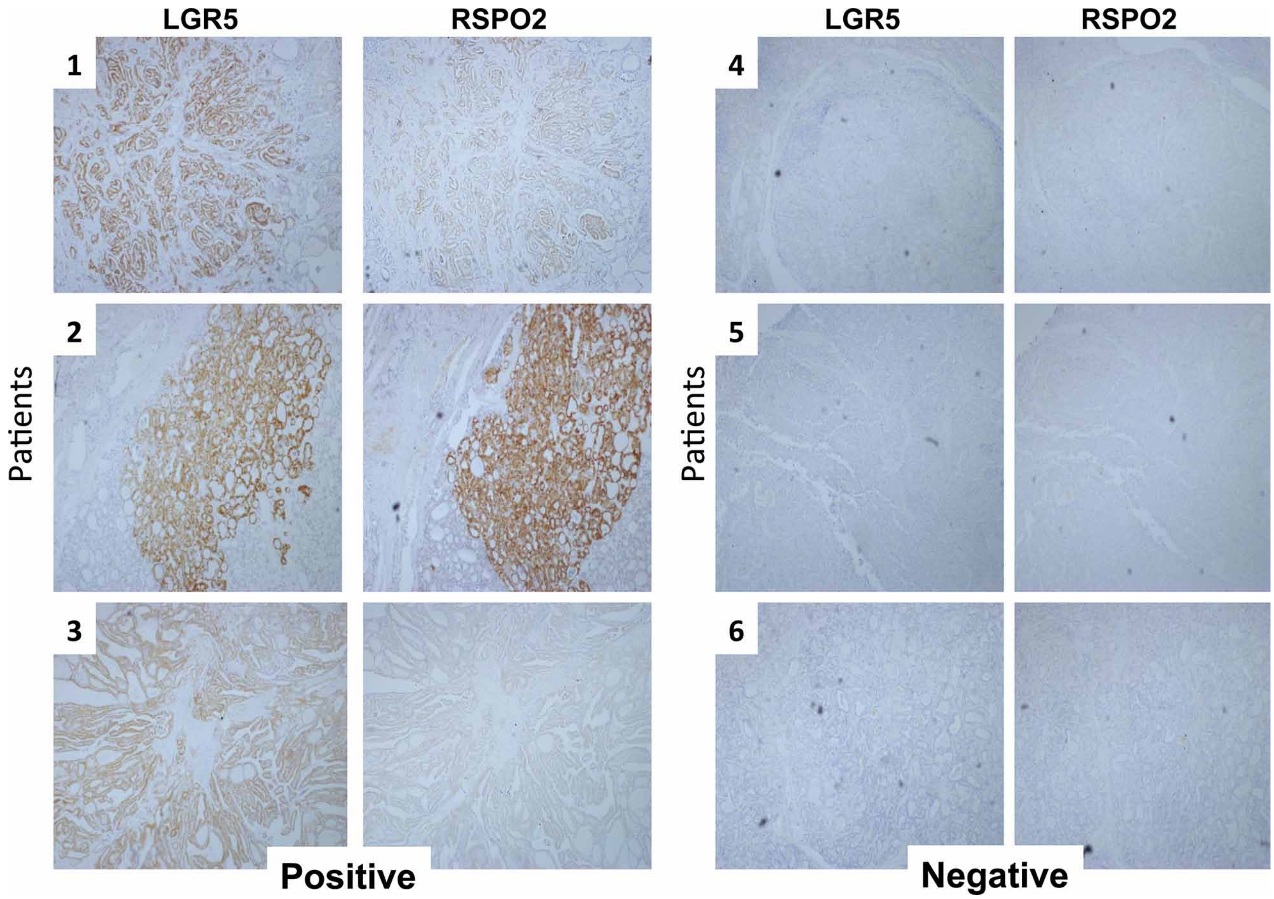

Supplementary Figure S2: LGR5 and RSPO2 stain similar tumor cell populations. Representative LGR5 and RSPO2 IHC staining from n = 6 patients with serial tumor sections demonstrates positive tumors (patients 1–3) and negative tumors (patients 4–6) (magnification x4). Similar areas of tumor stain either positive or negative for RSPO2 ligand and LGR5 receptor, respectively.

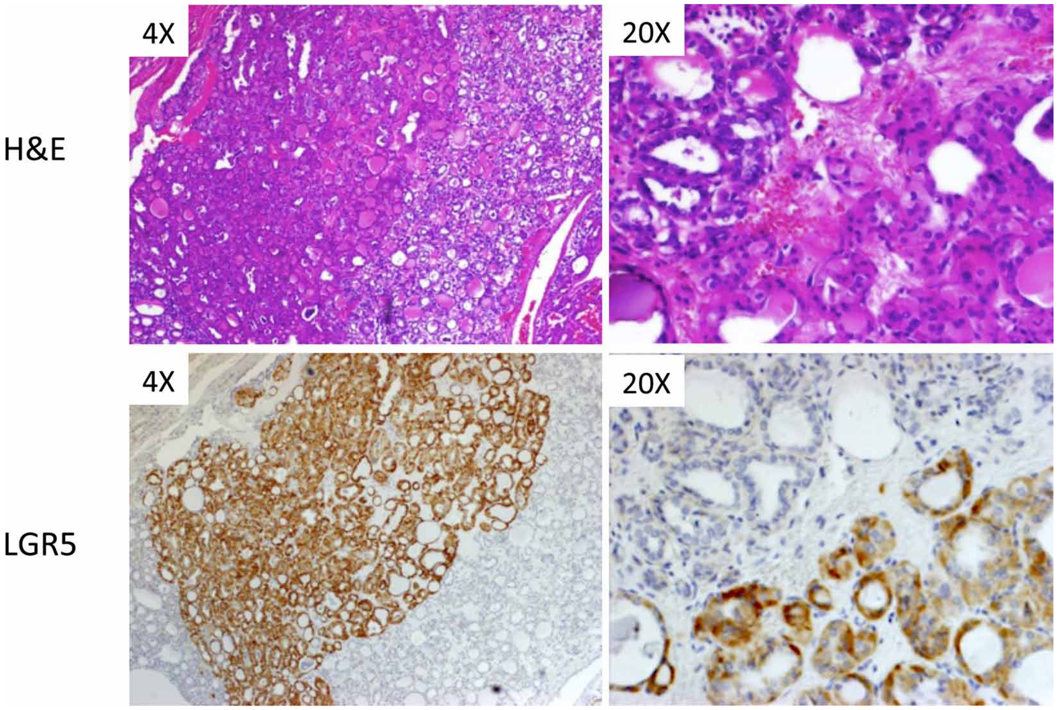

Supplementary Figure S3: LGR5 Intratumoral Heterogeneity. There can be tumor heterogeneity in human papillary thyroid cancer, with distinct LGR5+ and LGR5- populations of cells. Corresponding H&E and DAB-staining for LGR5 (magnification x4 and x20 shown).

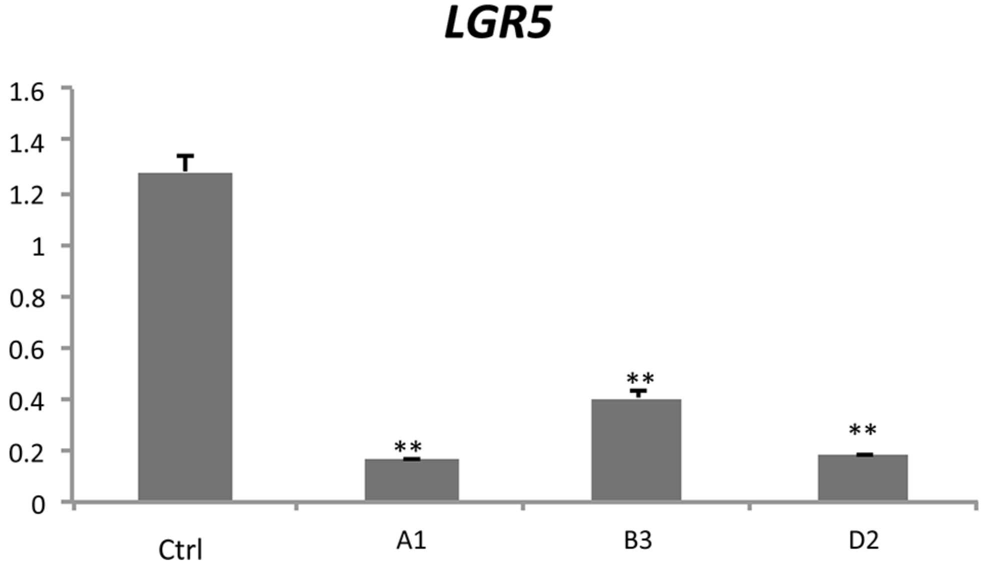

Supplementary Figure S4: Individual shRNA constructs suppress LGR5 expression. Three separate shRNA silencing sequences were utilized in the *in vitro* knockdown of LGR5 in the TPC-1 cell line (A1, B3, and D2). All constructs similarly suppressed *LGR5* expression, with A1 and D2 suppressing to approximately 15% of baseline levels. All three transfected constructs were transfected and behaved similarly in the scratch assay.

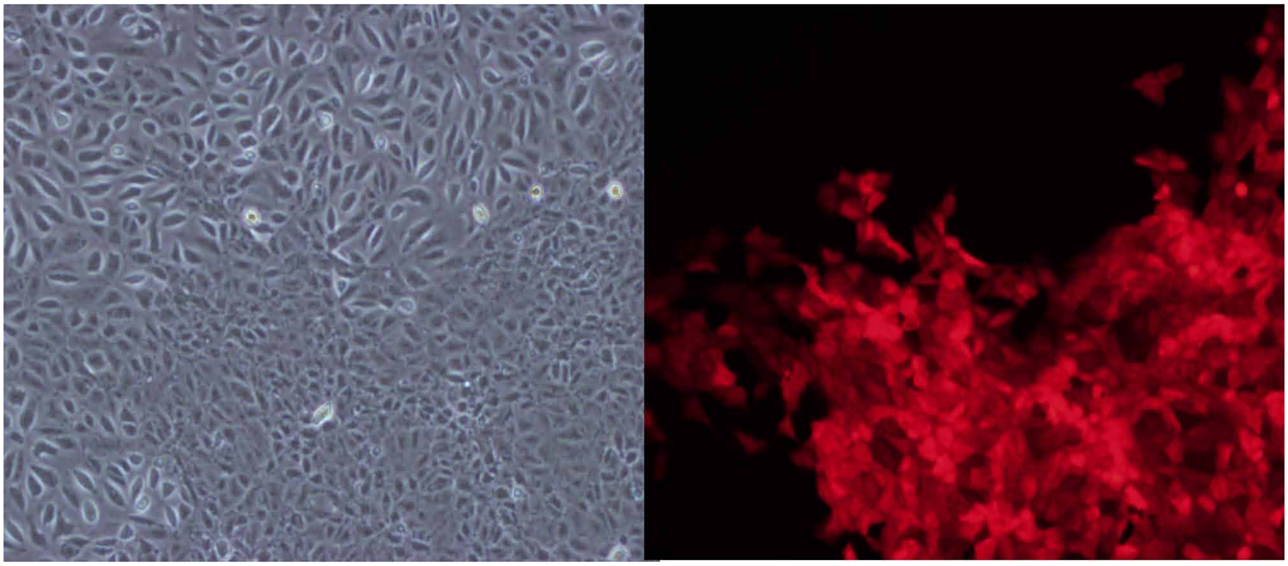

Supplementary Figure S5: Stable transfection of shRNA targeting LGR5 in human TPC-1 cell line. Demonstration of successfully transfected cells by shRNA shown by immunofluorescence in Cherry red (magnification x20). Transfected *LGR5* shRNA TPC-1 cell lines were grown out to confluence and used in cellular growth and migration assays as described in *Methods*.

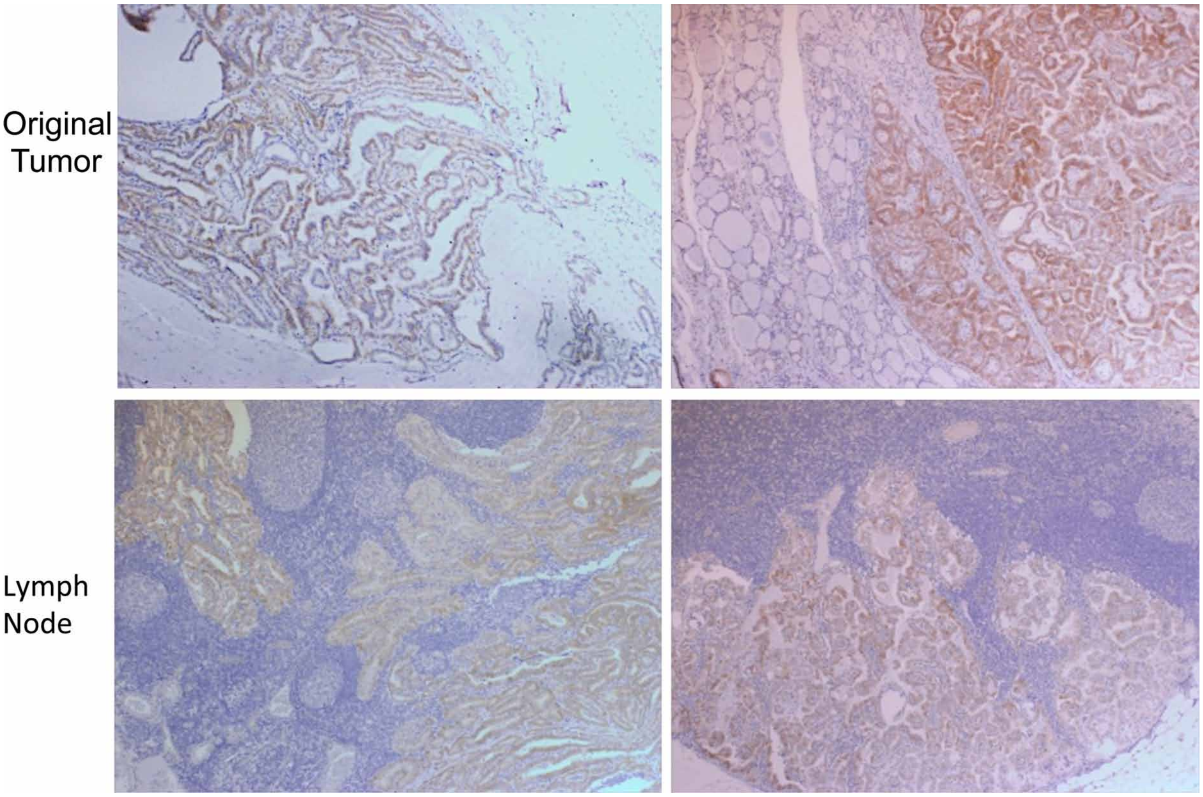

Supplementary Figure S6: LGR5 positive tumors were associated with LGR5 positive lymph nodes. Representative corresponding original tumors and lymph node metastatic disease. LGR5 staining by IHC is shown in brown, (magnification x4).

Supplementary Table S1: Primer Sequences

Human Primers	Forward	Reverse
Beta Actin	5′-TGGCATCCACGAAACTACCT-3′	5′-ACGGAGTACTTGCGCTCAG-3′
LGR5	5′-CTTCCAACCTCAGCGTCTTC-3′	5′-TTTCCCGCAAGACGTAACTC-3′
RSPO1	5′-TACTCAGTATTAAGGTTGG-3′	5′-CCTCGGAATATCATATGAG-3′
RSPO2	5′-GAATGTGTGGAAGGATG-3′	5′-GTGCGATTATTTCTGCTA-3′
RSPO3	5′-ATCCAGCAAAGAAATCC-3′	5′-GATACCGATTTCTGTTTATC-3′
Axin2	5′CTGCCACCAAGACCTACATAAG-3′	5′-GATAGCCACACACGACTTTAG-3′
Beta-Catenin	5′CTGCTGTTTTGTTCCGAATGTC-3′	5′CCATTGGCTCTGTTCTGAAGAGA-3′
